# Burnout and workplace dehumanization at the supermarket: A field study during the COVID‐19 outbreak in Italy

**DOI:** 10.1002/casp.2588

**Published:** 2021-11-29

**Authors:** Roberta Rosa Valtorta, Cristina Baldissarri, Chiara Volpato

**Affiliations:** ^1^ Department of Psychology University of Milano‐Bicocca Milano Italy

**Keywords:** burnout, COVID‐19, dehumanization, supermarket staff

## Abstract

This study explores the psychological effects of the COVID‐19 emergency on workers employed in the supermarket sector by analysing their levels of burnout and the relationship between the burnout syndrome and employees' workplace experiences. A sample of 422 Italian workers answered a survey addressing the burnout dimensions (i.e., exhaustion, cynicism, and professional inefficacy) along with perceived organizational factors and dehumanizing representations. Results showed that 32% of the respondents had symptoms of severe burnout, and 41% had symptoms of exhaustion and cynicism. More specifically, through cluster analysis, four burnout profiles were identified: “burnout” (high on all three dimensions), “engagement” (low on all three dimensions), “overextended” (high on exhaustion), and “disengaged” (moderate on exhaustion and cynicism). Each cluster showed a different pattern of correlates with the organizational and dehumanizing perceptions. Our findings contribute to the knowledge gaps of burnout and workplace experiences by providing insights into the ongoing health emergency among supermarket clerks. Please refer to the Supplementary Material section to find this article's Community and Social Impact Statement.

The outbreak of coronavirus disease (COVID‐19) that began in China has spread worldwide. In Italy, the Western country that first had to struggle with a massive outbreak of the virus, professionals who were involved in the care of patients with COVID‐19 reported frequent work‐related somatic symptoms, especially in terms of exhaustion (Barello, Palamenghi, & Graffigna, 2020). Similar results were reported by Giusti et al. (2020), according to whom moderate to severe levels of exhaustion and reduced professional efficacy were present in more than 60% of the Italian health professionals considered by the scholars. Even though these findings are important for the understanding of the psychological consequences of the COVID‐19 pandemic on healthcare practitioners, very little is known about the effects of this exceptional situation on other types of workers.

In March 2020, many areas of Italy began to close schools and businesses to prevent the spread of the virus. However, much of the food sector has continued operating. Supermarket clerks were unexpectedly pushed to the frontline of this pandemic, but they were often not prepared with adequate knowledge. In this regard, Ramaci, Pagliaro, Teresi, and Barattucci (2021) found that, during the COVID‐19 outbreak in Italy, high levels of job demands were negatively associated to job satisfaction and positively related to the fatigue and burnout perceived by supermarket clerks.

Despite these findings, no further research has examined the impact of working during the COVID‐19 crisis on supermarket staff in Italy, where the virus has infected over 4,717,899 people and claimed 131,541 lives.^1^ Thus, through a study conducted between March and April 2020—when Italy became the first Western country hit by the novel coronavirus and the lockdown was imposed—we aimed to investigate the psychological effects of this pandemic on supermarket workers in an explorative way by analysing their levels of burnout and the relationship between the burnout syndrome and employees' workplace experiences. More specifically, we aimed to first investigate the levels of burnout among supermarket clerks by examining the association of specific burnout profiles with several organizational factors, such as work overload, unsafe work environment, and supportive relationships, namely the traditional elements that have been studied in the burnout research (e.g., Maslach, 2001) and critically affected by the current pandemic. Moreover, for the first time in the literature, we aimed to explore the relationship between different burnout profiles and specific workplace dehumanizing representations.

## 1 BURNOUT SYNDROME AND ORGANIZATIONAL FACTORS

Burnout is a psychological syndrome characterized by the three dimensions of exhaustion, cynicism, and professional efficacy. Exhaustion is described as loss of energy and fatigue. Cynicism refers to an indifferent attitude towards work. Professional efficacy instead concerns feelings of successful achievement, and accomplishments for one's work (Maslach, Schaufeli, & Leiter, 2001).

Some authors proposed an innovative approach for studying burnout by showing that using multiple dimensions can allow the identification of distinct pattern, or profiles, of this syndrome according to the individual experience of work. For example, Leiter and Maslach (2016) highlighted five distinct profiles among healthcare employees: the “burnout” and the “engagement” profiles and three intermediate profiles, namely a “disengaged” profile (high cynicism, moderate scores on the other measures), an “overextended” profile (high exhaustion, moderate scores on the other measures), and an “ineffective” profile (high professional inefficacy, moderate scores on the other measures).

Within the context of this literature, there are various studies dealing with the links between burnout and several organizational factors, such as lack of control, insufficient reward, work overload, unsafe work environment, and supportive relationships (see Maslach, 2001). These last three components seem to be the most relevant during the current pandemic. Indeed, Morgantini et al. (2020) found that, among healthcare professionals working throughout the COVID‐19 pandemic, burnout is increased, and it is related to high workload and limited organizational support. Accordingly, Jalili, Niroomand, Hadavand, Zeinali, and Fotouhi (2021) speculated that heavy work overload, lack of staff support, and an unsafe workplace might result in a state of high burnout among frontline workers. These considerations echo those of previous studies. For example, some investigations conducted before the COVID‐19 pandemic showed that the excessive use of robotic service and standardized operations creates exhaustion and fatigue for service personnel (Leidner, 1993; Mattila & Enz, 2002), loss of self‐esteem, feelings of low self‐worth and increased employees' discomfort (Vella, Gountas, & Walker, 2009). Other research obtained results in this regard, showing that workers in the “first line” of contact with the client are more likely to experience burnout due to the high repetitiveness of their job, especially in terms of emotional exhaustion and cynicism (Johnson, Holdsworth, Hoel, & Zapf, 2013; Singh, 2000). Furthermore, Moreno‐Jiménez et al. (2004)'s investigation on burnout carried out with supermarket workers found that activities performed by cashiers have a strong emotional component. Indeed, the interaction with customers exceeds 80% of the time invested in their working day, requiring expressing positive emotions and controlling or inhibiting negative feelings. As reported by Colom and Contreras (2018), this continuous emotional management can create dissonance by thus undermining workers' well‐being and increasing the burnout syndrome.

Related to these findings, Tayfur and Arslan (2013) asserted that mismatches in workload might aggravate exhaustion by generating an anxiety condition. Furthermore, they showed that negative organizational factors (i.e., workload and time pressure) are related to burnout, particularly the exhaustion dimension. Empirical research (e.g., Nahrgang, Morgeson, & Hofmann, 2011) has found that also the perception of unsafe work environments evokes a health impairment process that exhausts employees' mental and physical resources. Workplace safety is a relevant factor that can contribute to exhaustion. Indeed, although some risks may be avoided by employees, the mere presence of hazards is likely to increase employees' perceptions of danger in the workplace and to be associated with psychological costs.

In addition, burnout researchers have investigated the quality of relationships with supervisors and co‐workers. Consiglio, Borgogni, Vecchione, and Maslach (2013) stated that the experience of unfairness at work fuels a sense of cynicism about the workplace and found an association between cynicism and teamwork. Furthermore, Leiter and Maslach (2016) showed that “disengaged” profiles of burnout (i.e., workers with high cynicism) were more characterized by distress regarding the community area of work‐life than “overextended” profiles (i.e., workers with high exhaustion).

Building from these arguments, we first examined the impact of the COVID‐19 outbreak on the psychological well‐being of Italian supermarket staff. Through cluster analysis, we aimed to categorize individuals according to their pattern of response on the scale measuring exhaustion, cynicism, and professional inefficacy (i.e., the reverse score of the professional efficacy dimension). Then, we investigated the relationships between burnout and workplace experiences by assuming that burnout profiles differed not solely on the clustering of the burnout scores, but on distinct patterns of workplace experiences. More specifically, we examined the extent to which employees' perceptions regarding organizational factors differed among the resulting burnout profiles. We hypothesized that workers with higher exhaustion would be more concerned with work overload and unsafe work environment than workers with lower ratings on this component (*Hypothesis* 1). In addition, we expected the workers with higher cynicism to show more negative scores on perceived supportive relationships with supervisors and co‐workers than those with lower levels of this burnout facet (*Hypothesis* 2). Importantly, we also assumed that the burnout profiles would reflect meaningful differences among employees' dehumanizing perceptions.

## 2 DEHUMANIZATION IN THE WORK DOMAIN

Dehumanization is a psychological process that refers to the idea that people are denied their humanness and can assume different forms, such as objectification and biologization, namely the consideration of others as more similar to objects or viruses rather than to human beings (Volpato & Andrighetto, 2015). Given the epidemic outbreak of the novel coronavirus and the extensive workload experienced by frontline workers in relation to this pandemic, we believe that these two forms of dehumanization might be particularly relevant to workers' well‐being. Indeed, the work overload together with a potentially unsafe work environment and the risk of contagion might make workers employed during this pandemic vulnerable to these specific dehumanizing perceptions.

Different research analysed the process of objectification in the work domain. For example, Andrighetto, Baldissarri, and Volpato (2017) revealed that workers performing subordinate activities were perceived as instrument‐like. Furthermore, Loughnan, Baldissarri, Spaccatini, and Elder (2017) found that merely recalling an objectifying work experience led employees to perceive themselves as less human. Evidence of the association between dehumanization and job features was provided by Valtorta, Baldissarri, Andrighetto, and Volpato (2019a), who found that occupations perceived as socially tainted (i.e., characterized by subordinate and repetitive tasks) are associated with an increased objectified perception of workers. In addition, the authors extended these findings by introducing the study of biologization. In their investigation, they demonstrated a link between occupations that involve a degrading work environment and biological dehumanization of workers. This relationship was replicated in experimental research (Valtorta, Baldissarri, Andrighetto, & Volpato, 2019b), in which the scholars showed that focusing on a degrading work environment increased feelings of disgust towards workers and, in turn, biologization towards them. Specifically, a low‐status occupation characterized by objectifying features and not characterized by a dirty work environment (i.e., the cashier) led to an objectified view of the target. Instead, a worker employed in a task associated with dirt (i.e., the janitor) was biologized and the results showed that this perception was related to the salience of a dirty work environment. Importantly, this occupation was less objectified than the cashier. Therefore, the scholars showed that while objectification seems to be related to the performed work activities, biologization seems to be associated with the work environment. Starting from this evidence, we believe that in the presence of work overload and risky work environments workers might be affected by objectification and biologization at the same time. Similarly, despite the lack of research especially on self‐biologization, it is plausible to think that during the current pandemic, also self‐dehumanizing perceptions in terms of instrument‐ and virus‐related metaphors can be experienced simultaneously by some workers.

Despite the relevance of these considerations, no further research has examined biologization in the work domain. Moreover, just a few studies have investigated the relationship between burnout and dehumanization. Vaes and Muratore (2013) found that by dehumanizing patients, health care workers can protect themselves against burnout and the emotional demands of working with suffering patients. Further evidence of the relationship between dehumanization and burnout was provided by research on organizational dehumanization that found that workers who perceived to be objectified by their organizations reported higher exhaustion (Caesens & Stinglhamber, 2019). In addition, Baldissarri, Andrighetto, and Volpato (2014) observed that supermarket workers' perception of being objectified by their supervisor was related to their tendencies to objectify themselves. Crucially, they found that this association was mediated by increased burnout. The above findings were also partially confirmed by Auzoult and Personnaz (2016), who analysed objectification among employees from various economic sectors (e.g., civil service, industry, health care) and demonstrated that the workers who perceived to be objectified by others had a tendency to self‐objectify.

Starting from the concept of burnout, we aimed to show the relationship of different burnout profiles with distinct workplace dehumanizing representations. Specifically, we considered the perception of being dehumanized by supervisors and customers and self‐dehumanization in terms of objectification and biologization. We first assumed that workers with higher exhaustion would report more objectifying perceptions and self‐perceptions than workers with lower ratings on this burnout component (*Hypothesis* 3). Our hypothesis is supported by previous research (e.g., Andrighetto et al., 2017), according to which subordinate work activities and severe workload conditions elicit objectifying processes of workers. Then, given that there is no previous research on burnout and biologization, we aimed to analyse this relationship in an exploratory way by supposing that workers with a full burnout profile (i.e., high exhaustion, cynicism, and professional inefficacy) would experience higher biologization than all the other workers. Indeed, biologization is one of the most extreme forms of dehumanization and has consistently emerged as a prominent correlate of degrading work environments and negative feelings towards workers (Valtorta et al., 2019a, 2019b). Therefore, it is plausible to imagine that perception of being biologized and self‐biologization would be higher among employees with higher exhaustion, cynicism, and inefficacy (*Hypothesis* 4). Most importantly, for the first time in the literature, we examined these relationships in a real work setting during a stressful event, such as a pandemic.

## 3 THE STUDY

### 3.1 Method

#### 3.1.1 Participants and procedure

Data were collected through a questionnaire using Qualtrics survey web‐system. Given the correlational nature of our study, we collected data on a large scale (i.e., *N* > 250). Indeed, as reported by Schönbrodt and Perugini (2013), this guarantees the stability of correlations. We considered an initial sample of 455 respondents. A sensitivity analysis conducted with G*Power (Faul, Erdfelder, Lang, & Buchner, 2007) showed that our sample was sufficient to detect small correlations (*r* = 0.13), assuming an α of 0.05 and power of 0.80. Some workers were recruited with the assistance of the senior human resources specialists of the participating supermarket companies. Other respondents were instead recruited from social network groups on Facebook.

To obtain a reliable sample, we included two attentional check items in our survey (e.g., “Please answer 3 to this item”) (see Oppenheimer, Meyvis, & Davidenko, 2009). Thirty‐three participants failed these items and were removed from the analyses. Thus, the final sample was composed of 422 Italian workers (346 females; *M*_age_ = 39.20, *SD* = 9.28; age range: 20–61) employed in 64 different supermarket chains. A second sensitivity analysis confirmed that this sample was sufficient to detect small correlations (*r* = 0.14).

#### 3.1.2 The survey

The order of presentation of the following scales was randomly varied and all the measures were introduced by the statement: “Please, respond to the following questions thinking about your work‐life experience during the novel coronavirus epidemic.”

Then, participants were asked to indicate some demographic information about themselves. They were finally debriefed and thanked for their participation.

##### Burnout

The Maslach Burnout Inventory‐General Scale (MBI‐GS; Schaufeli, Leiter, Maslach, & Jackson, 1996) was used to assess workers' levels of burnout during the COVID‐19 pandemic. The MBI‐GS is a three‐dimensional scale capturing exhaustion, cynicism, and professional efficacy. *Exhaustion* was measured with five items (e.g., “I feel burned out from my work”; *α* = .87). *Cynicism* was measured with five items (e.g., “I have become less enthusiastic about my work”; *α* = .77). *Professional efficacy* was measured with six items (e.g., “I feel I am making an effective contribution to what this organization does”; *α* = .71). Items were rated from 1 (*completely disagree*) to 7 (*completely agree*). The ratings for professional efficacy were reversed so that high scores reflected high professional inefficacy.

##### Organizational factors

Workers rated their perceptions about *work overload* and *unsafe work environment* by answering two questions: “Compared to how much you worked on average before the COVID‐19 emergency, how much has your workload increased during these weeks?” and “How adequate in terms of health precautions has your work environment been during these weeks?” on a scale from 0 (*not at all*) to 100 (*extremely*). The latter score was reversed, therefore higher ratings indicate negative perceptions of the work overload and work environment, respectively.

*Supportive relationships with supervisors* were measured with 11 items (e.g., “My supervisor genuinely cares about me”; *α* = .97) borrowed from Wu, Rusyidi, Claiborne, and McCarthy (2013). Workers were asked to express their agreement on a scale from 1 (*completely disagree*) to 7 (*completely agree*). Instead, the Inclusion of Other in the Self (IOS; Aron, Aron, & Smollan, 1992). Scale was used to measure perceived *supportive relationships with co‐workers*. Participants were asked to choose among seven pictures the one that best represented their relationship in terms of emotional closeness with their colleagues. Each image showed two circles (labelled “self” and “co‐workers”) with varying degrees of overlap, from nonoverlapping (i.e., 1) to almost completely overlapping (i.e., 7).

##### Perceptions of being dehumanized

To measure workers' perceptions of being objectified and biologized, participants were asked to express their agreement from 1 (*completely disagree*) to 7 (*completely agree*). More specifically, *perceptions of being objectified by supervisors* were assessed with nine statements (e.g., “The importance that my supervisor gives me depends entirely on my work skills”; *α* = .91) adapted from Baldissarri et al. (2014). Instead, *perceptions of being objectified and biologized by customers* ^2^ were assessed by using two scales created ad hoc and composed of seven and eight items, respectively. Example items were: “Customers at the supermarket where I work treat me like I am an object” for the perception of being objectified (*α* = .94) and “Customers at the supermarket where I work consider me as a virus to be kept at a distance” for the perception of being biologized (*α* = .86).^3^

##### Self‐dehumanizing perceptions

Self‐dehumanizing perceptions of the workers were measured by employing words that recalled objectification and biologization. Workers were asked to rate the extent to which they perceived themselves to be similar (1 = *not at all*, 7 = *extremely*) to six instrument‐related words (i.e., *instrument*, *tool*, *thing*, *machine*, *number*, *object*; *α* = .94) and five virus‐related words (i.e., *virus*, *contagion*, *disease*, *infection*, *epidemic*; *α* = .96) during their work activity (see Valtorta et al., 2019a).

### 3.2 Results

Table 1 reports descriptive statistics and correlations among variables. It is noteworthy that, among the three burnout components, work overload was positively associated with exhaustion, but not with cynicism and professional inefficacy. Further, the work overload score was positively correlated with self‐objectification, but it was unrelated to self‐biologization. Finally, perceived supportive relationships with supervisors and co‐workers were negatively associated with self‐objectification and self‐biologization.

**TABLE 1:** Descriptive statistics and correlations among variables

Variables	1	2	3	4	5	6	7	8	9	10	11	12
1. Exhaustion	—											
2. Cynicism	0.64^**^	—										
3. Professional inefficacy	0.26^**^	0.34^**^	—									
4. Work overload	0.17^**^	0.08	−0.03	—								
5. Unsafe work environment	0.33^**^	0.24^**^	0.15^**^	0.06	—							
6. Relationships with supervisors	−0.32^**^	−0.41^**^	−0.36^**^	−0.01	−0.25^**^	—						
7. Relationships with co‐workers	−0.20^**^	−0.32^**^	−0.24^**^	0.06	0.01	0.29^**^	—					
8. Objectified by supervisors	0.37^**^	0.36^**^	0.26^**^	0.06	0.25^**^	−0.64^**^	−0.25^**^	—				
9. Objectified by customers	0.31^**^	0.25^**^	0.17^**^	0.12^**^	0.17^**^	−0.16^**^	−0.14^**^	0.15^**^	—			
10. Biologized by customers	0.33^**^	0.28^**^	0.15^**^	0.08	0.21^**^	−0.16^**^	−0.09	0.18^**^	0.46^**^	—		
11. Self‐objectification	0.40^**^	0.37^**^	0.13^**^	0.17^**^	0.25^**^	−0.28^**^	−0.15^**^	0.33^**^	0.47^**^	0.24^**^	—	
12. Self‐biologization	0.25^**^	0.24^**^	0.12^**^	0.08	0.15^**^	−0.16^**^	−0.21^**^	0.21^**^	0.27^**^	0.39^**^	0.32^**^	—
*Mean*	4.72^a^	3.58	2.77	69.28^a^	60.75^a^	3.33	4.59^a^	4.56^a^	4.27^a^	3.52	4.55^a^	2.81
*SD*	1.68	1.48	1.01	26.14	24.89	1.69	1.99	1.60	1.71	1.41	1.83	1.90

^a^ Significantly higher than the mid‐point of the scale, *p* < .050.

*Note*: **p < .050.

#### 3.2.1 Cluster analysis

To identify the presence of distinct profiles on burnout variables, we examined their three‐dimensional array in cluster analysis. Then, we examined the distribution of respondents into various clusters and assessed differences in exhaustion, cynicism, and professional inefficacy ratings for each cluster.

**FIGURE 1:**
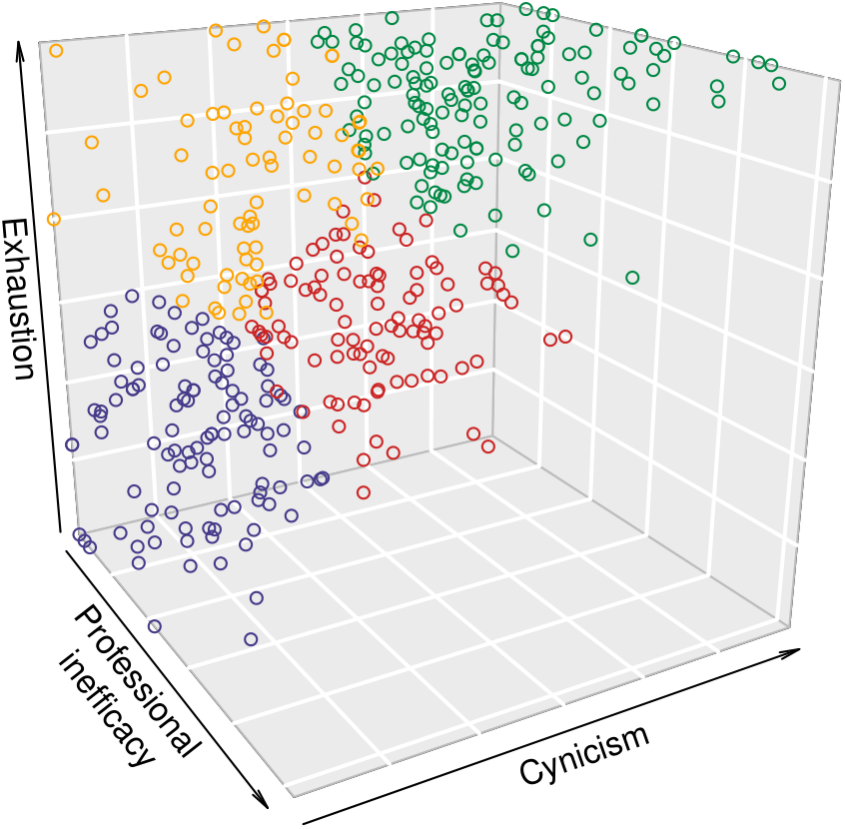
3‐D Scatterplot of exhaustion, cynicism, and professional inefficacy separated by cluster. The “burnout” cluster is plotted in green; the “engagement” cluster is plotted in blue; the “disengaged” cluster is plotted in red; the “overextended” cluster is plotted in yellow

To examine the three‐dimensional space structure, we conducted two types of cluster analysis. We first conducted a hierarchical cluster analysis (Ward's, 1963, method) to determine the best fitting number of clusters. We used the graphical scree plot technique as the stopping rule for determining the ideal number of clusters and, on this basis, we adopted a four‐cluster solution. We then conducted a *k*‐means cluster analysis (with the parallel threshold method) to determine which participants fit into which cluster. A 3D scatter plot of the data can be seen in Figure 1. Based on the patterns observed in this figure and previous literature (e.g., Leiter & Maslach, 2016), we labelled the four profiles as follows: (a) “burnout” (high exhaustion, cynicism, and professional inefficacy), (b) “engagement” (low exhaustion, cynicism, and professional inefficacy), (c) “disengaged” (moderate exhaustion and cynicism, low professional inefficacy), and (d) “overextended” (high exhaustion, low cynicism, and professional inefficacy).

Table 2 displays the scores for each of the burnout dimensions (i.e., exhaustion, cynicism, and professional inefficacy) for the four clusters. “Burnout,” “disengaged,” and “overextended” profiles comprised 73% of the sample among them. The “engagement” profile made up the remaining 27%.

**TABLE 2:** Means and standard deviations (in parentheses) for the burnout dimensions by cluster

		Burnout dimensions		
Cluster	N	Exhaustion	Cynicism	Professional inefficacy
Burnout	136	6.40_Aa_ (0.62)	5.14_Ab_ (0.91)	3.21_Ac_ (1.11)
Engagement	111	2.69_Ba_ (0.85)	1.98_Bb_ (0.60)	2.31_Bc_ (0.87)
Disengaged	98	3.97_Ca_ (0.65)	3.88_Ca_ (0.73)	2.97_Ab_ (0.81)
Overextended	77	5.64_Da_ (0.84)	2.73_Db_ (0.74)	2.42_Bc_ (0.82)

*Note*: Capital subscripts compare clusters within the three dimensions; small subscripts compare the three dimensions within clusters.

To assess differences in exhaustion, cynicism, and professional inefficacy ratings for each cluster, we conducted a repeated‐measures ANOVA with the three burnout components as a within‐subjects factor and the cluster membership as a between‐subjects factor. Results showed a main effect of burnout components, *F*(1,418) = 1,161.59, *p* < .001, *η*_*p*_^2^ = .74, and a main effect of cluster membership, *F*(3,418) = 539.02, *p* < .001, *η*_*p*_^2^ = .80. Furthermore, results showed a significant interaction effect burnout components × cluster membership, *F*(3,418) = 175.52, *p* < .001, *η*_*p*_^2^ = .56. As reported in Table 2, the cluster “burnout” significantly differed on the exhaustion and cynicism ratings from all the other clusters (all *ps* < .001). Regarding the professional inefficacy, the cluster “burnout” significantly differed from the cluster “engagement” and “overextended” (all *ps* < .001) but did not differ from the cluster “disengaged” (*p* = .055). Despite this nonsignificant difference, the cluster “burnout” was the one with the highest scores on all the three burnout dimensions. Comparing the scores on exhaustion, cynicism, and professional inefficacy of this cluster, pairwise comparisons with Bonferroni adjusted alpha levels showed a significant difference among all these ratings (all *ps* < .030).

The cluster “engagement” significantly differed on the exhaustion and cynicism scores from all the other clusters (all *ps* < .001). Regarding the professional inefficacy, the cluster “engagement” significantly differed from the cluster “burnout” and “disengaged” (all *ps* < .001), but there was a nonsignificant difference between this cluster and the cluster “overextended” (*p* = .403). Despite this result, the cluster “engagement” was the one with the lowest ratings on all the three burnout dimensions. Comparing the scores on exhaustion, cynicism, and professional inefficacy of this cluster, results revealed a significant difference among all these ratings (all *ps* < .004).

Comparing the scores on exhaustion, cynicism, and professional inefficacy of the cluster “disengaged,” pairwise comparisons with Bonferroni adjusted alpha levels revealed a nonsignificant difference between the exhaustion and cynicism ratings (*p* = .374). These scores were significantly higher than the professional inefficacy rating (all *ps* < .001).

Finally, comparing the scores on exhaustion, cynicism, and professional inefficacy of the cluster “overextended,” the analyses revealed a significant difference among all these ratings (all *ps* < .020). Despite these significant comparisons, it is important to note that the exhaustion rating in this cluster was substantially higher than the cynicism and professional inefficacy scores.

#### 3.2.2 Organizational factors

A MANOVA was conducted to analyse the difference among participants' perceptions of work overload, unsafe work environment, and supportive relationships with supervisors and co‐workers according to the four burnout profiles (i.e., “burnout” vs. “engagement” vs. “disengaged” vs. “overextended”). The multivariate test revealed a main effect of cluster membership. As reported below, univariate tests showed a significant effect of the cluster on perceived work overload, unsafe work environment, and supportive relationships with supervisors and co‐workers.

##### Work overload

**FIGURE 2:**
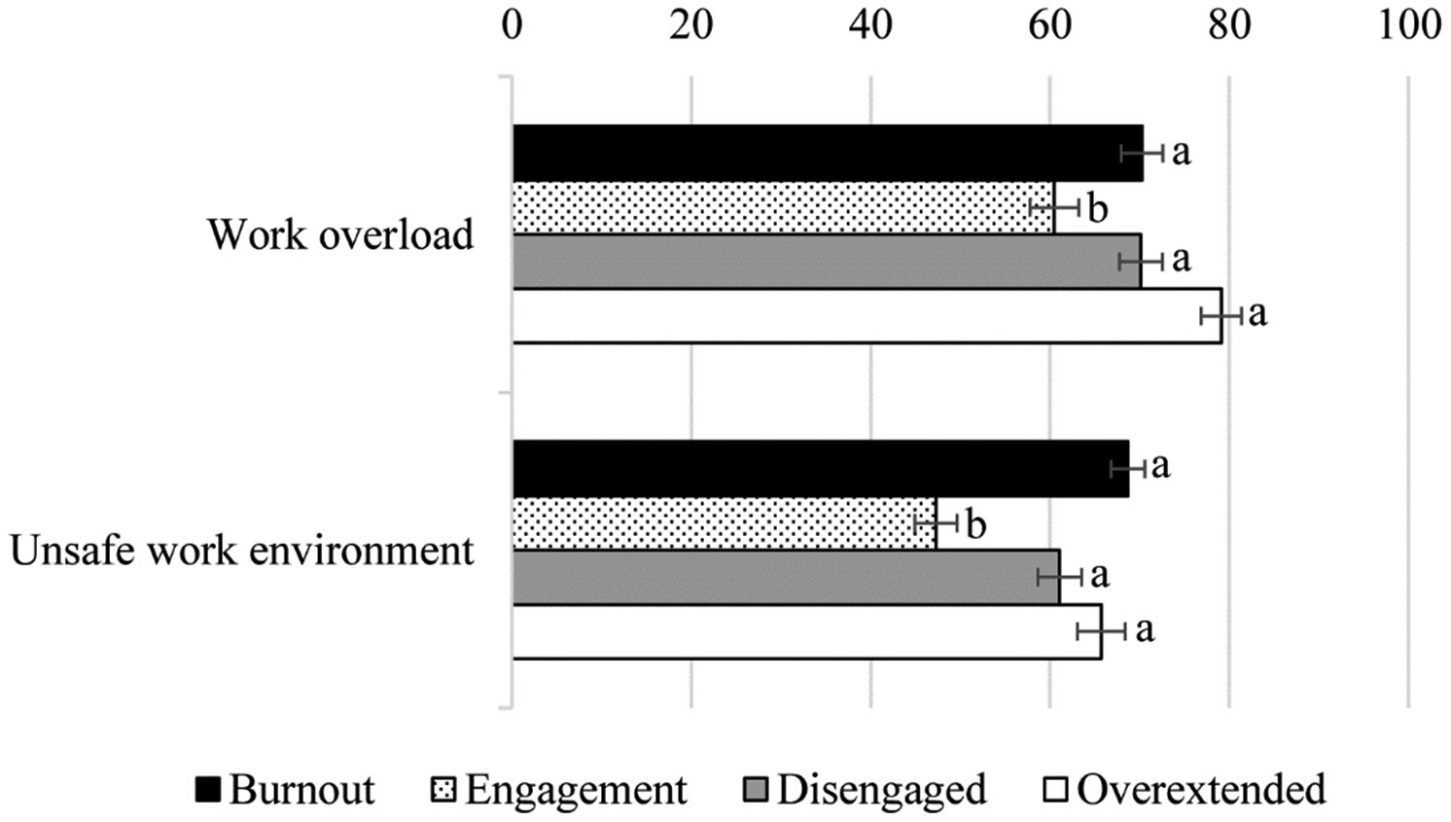
Perceived work overload and unsafe work environment as a function of the cluster membership. Bars with different letters within the same variable are significantly different, *p* < .050

As reported in Table 3, the analysis showed a significant effect of cluster membership, indicating that the “overextended” profile had a more negative view of the workload than the “engagement” profile, *p* < .001, but was not significantly different from the “burnout” profile, *p* = .091, and the “disengaged” profile, *p* = .128. The latter two did not significantly differ each other, *p* = 1.00, but both had a more negative perceptions than the “engagement” profile, all *ps* < .040 (see Figure 2).

**TABLE 3:** Differences among burnout clusters on organizational factors and dehumanizing perceptions

		Cluster						
		Burnout	Engagement	Disengaged	Overextended			
Variables	Range	*M* (*SD*)	*M* (*SD*)	*M* (*SD*)	*M* (*SD*)	*F* (3,418)	*p*	*η*_*p*_^2^
Work overload	0–100	70.26 (27.00)	60.49 (28.51)	70.14 (23.61)	79.12 (19.74)	8.33	< .001	0.06
Unsafe work environment	0–100	68.69 (21.88)	47.28 (24.84)	61.08 (24.14)	65.73 (23.28)	18.54	< .001	0.12
Relationships with supervisors	1–7	2.54 (1.37)	4.18 (1.74)	3.07 (1.44)	3.80 (1.71)	26.05	< .001	0.16
Relationships with co‐workers	1–7	3.99 (1.99)	5.32 (1.83)	4.21 (2.00)	5.06 (1.77)	12.58	< .001	0.08
*Note*: Multivariate *λ* = .69, *F*(6,834) = 13.62, *p* < .001, *η*_*p*_^2^ = .12								
Objectified by supervisors	1–7	5.13 (1.49)	3.68 (1.59)	4.61 (1.43)	4.76 (1.50)	19.69	< .001	0.12
Objectified by customers	1–7	4.76 (1.77)	3.68 (1.59)	4.20 (1.57)	4.65 (1.42)	14.94	< .001	0.10
Biologized by customers	1–7	4.15 (1.45)	3.01 (1.37)	3.25 (1.03)	3.49 (1.44)	16.65	< .001	0.11
*Note*: Multivariate *λ* = .77, *F*(9,1,012.56) = 13.03, *p* < .001, *η*_*p*_^2^ = .09								
Self‐objectification	1–7	5.24 (1.62)	3.33 (1.82)	4.68 (1.57)	4.91 (1.64)	29.03	< .001	0.17
Self‐biologization	1–7	3.41 (2.07)	2.27 (1.67)	2.67 (1.67)	2.70 (1.95)	8.19	< .001	0.06
*Note*: Multivariate *λ* = .81, *F*(6,834) = 15.51, *p* < .001, *η_p_* ^2^ = .10.								

##### Unsafe work environment

A similar pattern of results emerged for work environment. Pairwise comparisons with Bonferroni adjusted alpha levels showed that the “burnout,” “overextended,” and “disengaged” profiles had more negative views of the work environment than the “engagement” profile, all *ps* < .001. The former three profiles did not significantly differ each other, all *ps* > .090 (see Table 3 and Figure 2).

##### Supportive relationships with supervisors and co‐workers

**FIGURE 3:**
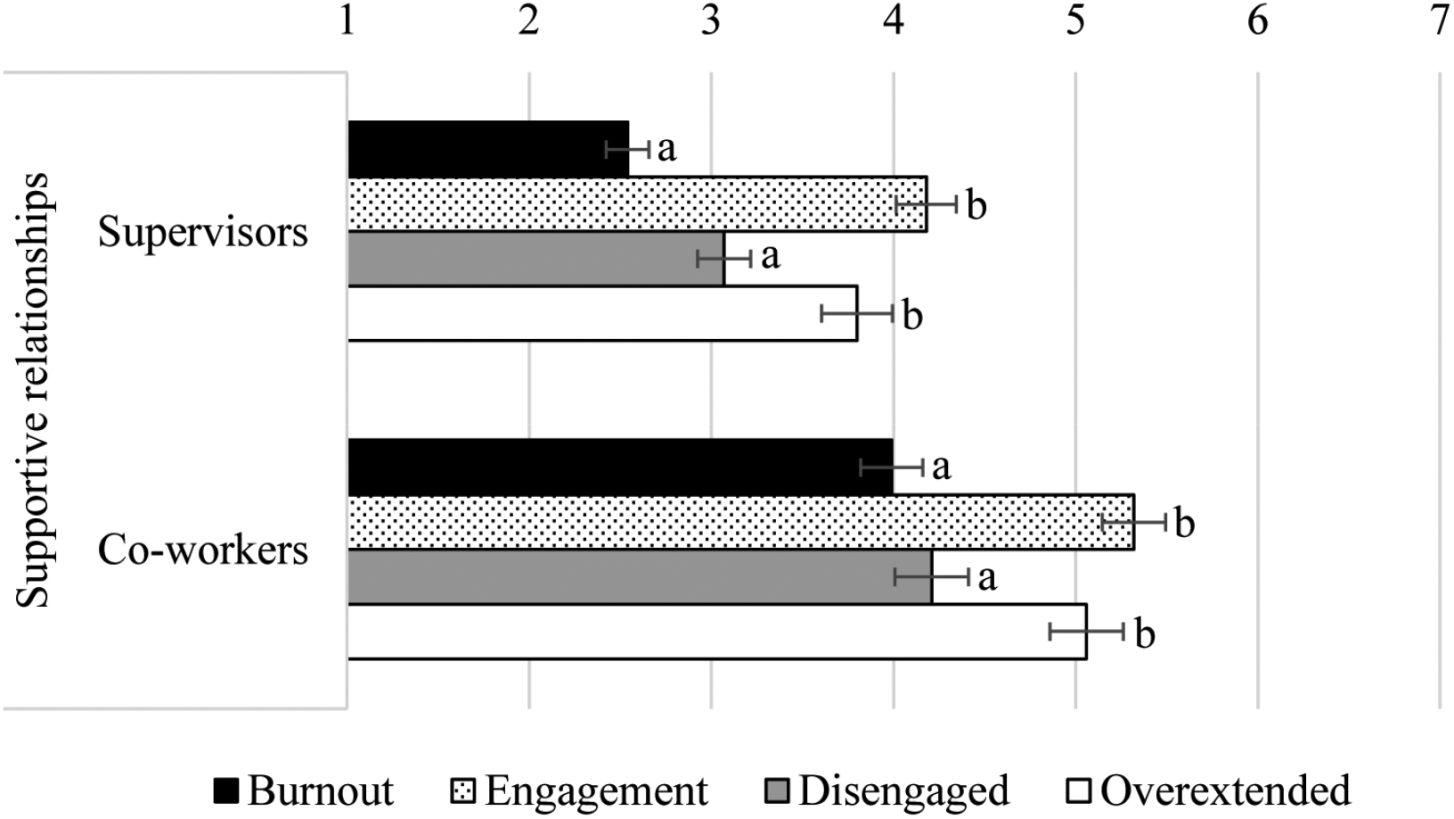
Supportive relationships with supervisors and co‐workers as a function of the cluster membership. Bars with different letters within the same variable are significantly different, *p* < .050

Regarding perceived relationships with supervisors, means, from most to least negative, were “burnout,” “disengaged,” “overextended,” and “engagement.” Pairwise comparisons with Bonferroni adjusted alpha levels demonstrated that means for supportive relationships with supervisors in the “burnout” and “disengaged” groups were more negative than the “overextended” and “engagement” groups, all *ps* < .013. Furthermore, the “burnout” and “disengaged” profiles did not significantly differ, *p* = .062. Similarly, the difference between the “overextended” group and the “engagement” group was not significant, *p* = .603 (see Table 3 and Figure 3).

The same pattern of results emerged for perceived supportive relationships with co‐workers. Means for supportive relationships with colleagues in the “burnout” and “disengaged” profiles did not significantly differ, *p* = 1.00, and were more negative than those reported by the “overextended” and “engagement” groups, all *ps* < .022. The difference between the latter two groups was not significant, *p* = 1.00 (see Table 3 and Figure 3).

In line with our hypotheses, we found that the cluster membership was associated with different perceptions of specific organizational factors. In particular, we found that workers with higher exhaustion during the COVID‐19 pandemic (i.e., “burnout,” “overextended,” and “disengaged” profiles) had more negative views of the work overload and work environment than workers with lower levels of all the three burnout components (i.e., the “engagement” group; *Hypothesis* 1). Furthermore, results showed that workers with higher levels of cynicism during the COVID‐19 pandemic (i.e., “burnout” and “disengaged” profiles) were more negative on perceptions related to supportive relationships with supervisors and co‐workers than were other workers (*Hypothesis* 2).

#### 3.2.3 Perceptions of being dehumanized

A MANOVA was then conducted to analyse the effect of the burnout clusters (i.e., “burnout” vs. “engagement” vs. “disengaged” vs. “overextended”) on workers' perceptions of being dehumanized. The multivariate test revealed a main effect of cluster membership. As reported below, univariate tests showed a significant effect of the cluster on each dehumanization score.

##### Perceptions of being objectified by supervisors and customers

**FIGURE 4:**
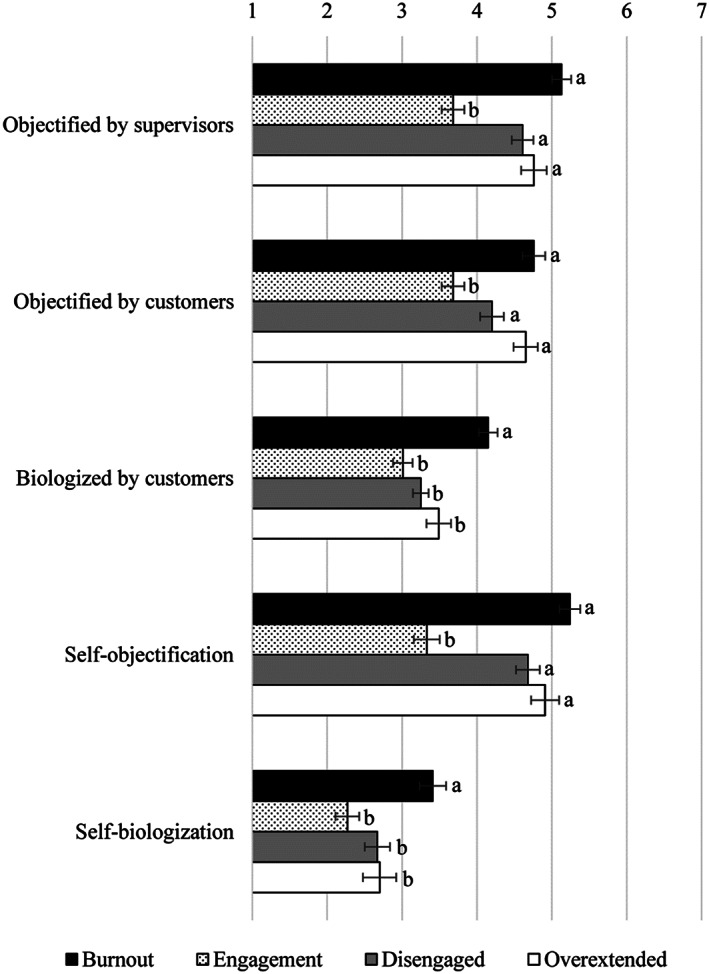
Perceptions of being dehumanized by supervisors and customers and self‐dehumanization as a function of the cluster membership. Bars with different letters within the same variable are significantly different, *p* < .050

The “burnout” profile had a higher perception of being objectified by the manager than the “engagement” profile, *p* < .001, but was not significantly different from the “overextended” profile, *p* = .524, and the “disengaged” profile, *p* = .058. The latter two did not significantly differ each other, *p* = 1.00, but both had a more objectifying perception than the “engagement” profile, all *p*s < .001 (see Table 3 and Figure 4).

A similar pattern of results emerged for perceptions of being objectified by customers. As reported in Table 3, pairwise comparisons with Bonferroni adjusted alpha levels demonstrated that means in the “burnout,” “overextended,” and “disengaged” groups were higher than the “engagement” profile, all *ps* < .007. Furthermore, the perceptions of being objectified by customers reported by workers in the “burnout,” “overextended,” and “disengaged” groups did not significantly differ, all *ps* > .060 (see Figure 4).

##### Perceptions of being biologized by customers

The workers in the “burnout” group reported a higher perception of being biologized by customers than workers in the “overextended,” “disengaged,” and “engagement” groups, all *ps* < .005. The latter three profiles did not significantly differ each other, all *ps* > .090 (see Table 3 and Figure 4).

In line with our expectations, results showed that workers with higher levels of exhaustion during the emergency due to the COVID‐19 spread (i.e., “burnout,” “overextended,” and “disengaged” profiles) reported greater perceptions of being objectified by supervisors and customers than workers with lower levels of all the three burnout components (i.e., the “engagement” group; *Hypothesis* 3). Instead, only workers in the “burnout” group reported significantly higher perceptions of being biologized by customers than all the other workers (*Hypothesis* 4).

#### 3.2.4 Self‐dehumanizing perceptions

A MANOVA confirmed the relationship of self‐dehumanizing perceptions with clusters. As reported below, univariate tests showed a significant effect of the cluster on each self‐dehumanization score.

##### Self‐objectification

The “burnout” profile reported a higher self‐objectification than the “engagement” profile, *p* < .001, but was not significantly different from the “overextended” profile, *p* = 1.00, and the “disengaged” profile, *p* = .066. The latter two did not significantly differ each other, *p* = 1.00, but both had a more self‐objectifying perception than the “engagement” profile, all *ps* < .001 (see Table 3 and Figure 4).

##### Self‐biologization

As reported in Table 3, the analysis showed a significant effect of cluster membership, indicating that the workers in the “burnout” group reported a higher self‐biologization than workers in the “overextended,” “disengaged,” and “engagement” groups, all *ps* < .050. The latter three profiles did not significantly differ each other, all *ps* > .700 (see Figure 4).

As hypothesized, these findings revealed that workers with higher exhaustion during the COVID‐19 pandemic (i.e., “burnout,” “overextended,” and “disengaged” profiles) self‐objectified more than workers in the “engagement” group (*Hypothesis* 3). In this regard, our results seem to confirm the literature on workplace objectification (e.g., Baldissarri et al., 2014; Caesens, Stinglhamber, Demoulin, & De Wilde, 2017) by showing that workers more concerned with work overload, and thus emotionally exhausted, also reported higher perceptions of being objectified and internalized this representation. Instead, regarding biologization, results showed that workers in the “burnout” group self‐biologized more than all the other workers (*Hypothesis* 4).

## 4 DISCUSSION

The main aim of this study was to highlight the psychological impact of the COVID‐19 emergency on Italian workers employed in the supermarket sector. In particular, our purpose was to investigate the levels of burnout among supermarket clerks and the extent to which employees' workplace experiences differed among burnout profiles. We provided evidence for the assumptions that different levels of burnout are associated with distinct workplace experiences in terms of organizational factors and dehumanizing representations. Specifically, among Italian workers employed in supermarkets during the COVID‐19 pandemic, our findings revealed the presence of two endpoint profiles of “burnout” and “engagement,” as well as two intermediate profiles of “disengaged” and “overextended.” The two intermediate profiles, which displayed a pattern of a high score on exhaustion and moderate on exhaustion and cynicism, were less negative than the “burnout” profile but more negative than the positive endpoint of “engagement.” Indeed, “burnout” emerged as the most negative profile with respect to all our variables (i.e., perceived organizational factors and dehumanizing representations). However, statistical comparisons showed that “disengaged” and “overextended” did not significantly differ from “burnout” in terms of work overload, unsafe work environment, perceptions of being objectified by supervisors and customers, and self‐objectification, but they significantly differed from the “burnout” group in terms of perceptions of being biologized by customers and self‐biologization. Finally, it is important to note that “disengaged” emerged as the most negative profile with respect to supportive relationships with supervisors and co‐workers. Thus, there was support for our hypotheses.

Unlike previous research showing that exhaustion is the strongest element of burnout (e.g., Toppinen‐Tanner, Kalimo, & Mutanen, 2002), our analyses revealed that, among workers employed in supermarkets during stressful times, exhaustion alone cannot be considered the most relevant component of burnout. We found indeed that the “burnout” profile was associated with a more negative work‐life experience than was the experience of exhaustion alone (i.e., the “overextended” profile). Furthermore, the “disengaged” profile (i.e., the combination of exhaustion and cynicism) appeared to be overall more negative than the “overextended” profile, suggesting that exhaustion per se is not a sufficient proxy for burnout. As stated by Leiter and Maslach (2016), the three dimensions of exhaustion, cynicism, and inefficacy are not so highly correlated as to constitute a one‐dimensional construct. Accordingly, four burnout profiles emerged from our study, and each of them showed a different pattern of correlates with organizational and social‐psychological variables. Workers with higher levels of cynicism during this pandemic (i.e., “burnout” and “disengaged” clusters) were more negative on perceptions related to supportive relationships than were other workers. Furthermore, those with higher exhaustion (i.e., “burnout,” “overextended,” and “disengaged” clusters) had more negative views of work overload and unsafe work environment than workers with lower levels of all the three burnout dimensions. A similar pattern of results emerged for perceptions of being objectified by supervisors and co‐workers and self‐objectification. Finally, we found that only workers in the “burnout” group reported significantly higher perceptions of being biologized and self‐biologization than all the other workers.

We believe that our study makes a novel contribution in different ways. First, our findings constitute one of the first empirical evidence of the presence of burnout among supermarket employees during the COVID‐19 outbreak in Italy. It is noteworthy that whereas existing research with frontline workers during health crises has been conducted almost exclusively with healthcare professionals (e.g., Barello et al., 2020; Giusti et al., 2020), we focused here on Italian supermarket clerks, namely workers who were unexpectedly pushed to the frontline of this pandemic. If we observe the overall mean ratings for all the considered variables (see Table 1), our findings show that perceived work overload, unsafe work environment, exhaustion, objectifying, and self‐objectifying representations exceeded the mid‐point of the respective response scales (i.e., 50 for work overload and unsafe work environment; 4 for all the other variables) by confirming our assumption that difficult working conditions during these stressful times have not been limited to healthcare settings. Indeed, all types of frontline workers faced risks with regard to their health and the possibility of potentially infecting their loved one. In line with this consideration, a national poll conducted in April 2020 by Eagle Hill Consulting found that 45% of US essential workers reported burnout. A more severe result emerged in August 2020, when the percentage reached 58%. We confirmed these alarming results and showed the presence of different patterns of correlates between distinct burnout profiles and organizational variables. Despite these considerations, 111 respondents who partecipated in the present research reported low scores on exhaustion, cynicism, and professional inefficacy. In this regard, it is worthwhile noting that the engagement profile showed higher scores on relationships with both supervisors and co‐workers. Thus, a supportive work context might have played a crucial role in protecting workers against burnout and increasing workplace well‐being, especially under the current difficult circumstances. Furthermore, it is important to note that we do not have data about burnout and engagement among these workers before the pandemic. Therefore, we do not know how respondents felt in regular times. In addition, although we did not collect data on job roles, contracts, and job insecurity in our research, it is plausible to think that workers with low‐status job roles reported lower levels of well‐being because of potential negative effects deriving from their position within the organization. Literature suggests indeed that perceptions of job insecurity might have negative consequences for low‐status employee attitudes (Rosenblatt, Talmud, & Ruvio, 1999), increase in job dissatisfaction (Davy, Kinicki, & Scheck, 1997), an increase in negative health outcomes (Hellgren & Sverke, 2003; Mohren, Swaen, van Amelsvoort, Borm, & Galama, 2003), and higher reports of psychological distress (Dekker & Schaufeli, 1995; Probst, 2000). Vice versa, in a sample of schoolteachers, Feather and Rauter (2004) showed that permanently employed workers who had secure jobs reported higher well‐being, stronger affective and emotional commitment to their school than contract teachers, who also reported higher feelings of insecurity, perceptions of little influence or control over their role‐related duties. In the present study, it is thus plausible that other factors beyond positive relationships, such as job roles, contracts, and job insecurity perceptions, might have protected some workers from the detrimental impact of the COVID‐19 pandemic and the deriving work overload.

Furthermore, our results expand research about dehumanization in the work domain. It has been found that subordinated jobs or critical task features (Andrighetto et al., 2017) are strongly associated with objectification and self‐objectification of workers. In addition, recent literature (e.g., Valtorta et al., 2019b) has consistently demonstrated that degrading work environments are associated with biological dehumanization. Our research adds a tile to this picture by providing the first empirical evidence of the relationships among distinct burnout states during stressful times and perceptions of being dehumanized and self‐dehumanization. More specifically, we found that workers with higher levels of exhaustion reported greater perceptions of being objectified by supervisors and customers and self‐objectification than employees with lower levels of all three burnout dimensions. Instead, workers with a full burnout profile reported greater perceptions of being biologized by customers and self‐biologization than all the other workers. Thus, whereas workplace objectification seems to be especially associated with loss of energy and fatigue, biologization shows a more complex picture, in which only respondents who experienced high exhaustion, cynicism, and inefficacy during the current pandemic reported greater perceptions to be seen as contagious entities by others and internalized this point of view. These findings are particularly relevant not only for the literature on dehumanization, but also for their potential consequences on personnel's well‐being. Research on objectification has shown that this dehumanizing perception can have detrimental consequences on workers, such as dismissing free will, increasing conforming behaviours or worsening task engagement and performance (e.g., Andrighetto et al., 2018; Baldissarri & Andrighetto, 2021; Baldissarri, Andrighetto, Gabbiadini, & Volpato, 2017). Moreover, our findings show that experiencing objectification and self‐objectification during these stressful times is associated with workers' feelings of being more exhausted, less independent, and engaged in their work by thus encouraging lower levels of productivity. Regarding biological dehumanization, no evidence has been documented on its consequences. However, considering the resulted association between biologization and the full burnout profile, it is plausible to imagine that workers who face this dehumanizing perception not only feel exhausted, but also less socially included in the workplace and experience greater emotional and cognitive distance from the work.

In addition, we believe that our findings have implications for future interventions also beyond this epidemic. For example, the pattern of association for workers with high exhaustion shows that one of their main issues is work overload. Therefore, the most relevant interventions for addressing this problem would be strategies to manage workplace demands, also because scholars have documented the serious impact of burnout deriving from work overload on job satisfaction and the intention of quitting the job (Srivastava & Agrawal, 2020). Instead, the primary concerns of workers with a “disengaged” profile seem to focus on positive relationships, so effective interventions would need to address these problems. In this regard, we believe that a supportive work context might also play a crucial role in protecting workers against workplace dehumanization, as suggested by the negative correlations between supportive relationships and dehumanizing and self‐dehumanizing perceptions that were found in the present study. Moreover, Caesens et al. (2017) found that higher organizational support increase job satisfaction by reducing the perception of being instrumentally dehumanized by the organization. These considerations seem to be consistent with previous research, according to which perceived organizational support is an important job resource that can help in achieving job demands and reducing turnover intention (see, for example, Srivastava & Agrawal, 2020). In particular, studies done on the association between supportive relationships and turnover intention have established that with high perceived support, the turnover intention among employees will reduce (Dawley, Houghton, & Bucklew, 2010; Islam, Ali, & Ahmed, 2018; Li, Bonn, & Ye, 2019), and they will be more willing to perform with higher risk (Neves & Eisenberger, 2014).

Despite the novelty of our study, some limitations should be considered. First, while sufficiently large and robust, the present sample is mainly composed of female workers (346 out of 422 respondents). Further investigations could increase the generalizability of our findings by using a more balanced sample across gender.

Furthermore, the present study is limited by its cross‐sectional and correlational perspectives, and as such cannot determine causality with regard to the variables analysed. Future research should examine the long‐term impact of the pandemic through a longitudinal design.

## 5 CONCLUSIONS

The present research suggests that the COVID‐19 pandemic has had an overwhelming impact on Italian supermarket staff. With about 32% of the respondents having symptoms of severe burnout and 41% with symptoms of exhaustion and cynicism, we can consider that the coronavirus outbreak has generated a mental health emergency in this occupational group. It is noteworthy that burnout emerged as associated with one of the most demanding psychological phenomena in the work domain, namely self‐dehumanization. Our results offer insights into the correlates of burnout, perceived organizational factors, and dehumanization of frontline workers during the acute phase of the COVID‐19 pandemic. We hope that our findings and future investigations encourage social‐psychological and organizational research to join efforts in order to manage potential future societal disasters where we will again rely on the efforts of workers to keep our societies afloat.

## CONFLICTS OF INTEREST

The authors declare that there is no conflict of interest.

## ETHICS STATEMENT

This research was conducted after receiving the ethical approval from the local Commission of the Department of Psychology for minimal risk study (Approval no. RM‐2020‐272). All procedures performed in the studies were in accordance with the APA ethical guidelines, the ethical principle of the Helsinki Declaration, and the Oviedo Convention on Human Rights and Biomedicine. At the beginning of the study, participants were informed about how the data were collected, processed, and stored. Full informed consent was obtained before participants started the survey.

## Supporting information


**Data S1.** Supporting information.

**Data S2.** Supporting information.

## Data Availability

Data and supplementary material are available through the Open Science Framework (https://osf.io/29mqa/?view_only=4cece1cac87e44cf950c26b115395f37).

## Endnotes

^1^
All the statistics were retrieved from https://covid19.who.int/ (last accessed on October 19, 2021).

^2^
For biologization, we did not measure perceptions of being biologized by supervisors, but only perceptions of being biologized by customers. Indeed, whereas workplace objectification has been frequently described as referring to hierarchical work relationships and implies others' perception as useful tools for one's purposes (Baldissarri et al., 2014), workplace biologization has been mainly analysed by examining laypeople's perception of certain occupational groups (Valtorta et al., 2019a). Therefore, unlike objectification, we believe that biologization can hardly be experienced by workers within the relationship with their supervisors as they belong to the same potentially unsafe work environment that, so far, has been found to be the main trigger of this kind of dehumanizing perception.

^3^
For the complete scales, see supplementary material on OSF, https://osf.io/29mqa/?view_only=4cece1cac87e44cf950c26b115395f37.

## References

[casp2588-bib-0001] Andrighetto, L. , Baldissarri, C. , Gabbiadini, A. , Sacino, A. , Valtorta, R. R. , & Volpato, C. (2018). Objectified conformity: Working self‐objectification increases conforming behavior. Social Influence, 13, 78–90. 10.1080/15534510.2018.1439769

[casp2588-bib-0002] Andrighetto, L. , Baldissarri, C. , & Volpato, C. (2017). (Still) modern times: Objectification at work. European Journal of Social Psychology, 47, 25–35. 10.1002/ejsp.2190

[casp2588-bib-0003] Aron, A. , Aron, E. N. , & Smollan, D. (1992). Inclusion of other in the self scale and the structure of interpersonal closeness. Journal of Personality and Social Psychology, 63, 596–612. doi:10.1037/0022-3514.63.4.596

[casp2588-bib-0004] Auzoult, L. , & Personnaz, B. (2016). The role of organizational culture and self‐consciousness in self‐objectification in the workplace. TPM: Testing, psychometrics, methodology. Applied Psychology, 23(3), 1–14. 10.4473/TPM23.3.1

[casp2588-bib-0005] Baldissarri, C. , & Andrighetto, L. (2021). Being treated as an instrument: Consequences of instrumental treatment and self‐objectification on task engagement and performance. Human Performance, 34, 85–106. 10.1080/08959285.2021.1878182

[casp2588-bib-0006] Baldissarri, C. , Andrighetto, L. , Gabbiadini, A. , & Volpato, C. (2017). Work and freedom? Working self‐objectification and belief in personal free will. British Journal of Social Psychology, 56, 250–269. 10.1111/bjso.12172 27862021

[casp2588-bib-0007] Baldissarri, C. , Andrighetto, L. , & Volpato, C. (2014). When work does not ennoble man: Psychological consequences of working objectification. TPM: Testing, Psychometrics, Methodology in Applied Psychology, 21, 327–339. 10.4473/TPM21.3.7

[casp2588-bib-0008] Barello, S. , Palamenghi, L. , & Graffigna, G. (2020). Burnout and somatic symptoms among frontline healthcare professionals at the peak of the Italian COVID‐19 pandemic. Psychiatry Research, 290, 113–129. 10.1016/j.psychres.2020.113129 PMC7255285 32485487

[casp2588-bib-0009] Caesens, G. , & Stinglhamber, F. (2019). The relationship between organizational dehumanization and outcomes: The mediating role of emotional exhaustion. Journal of Occupational and Environmental Medicine, 61, 699–703. 10.1097/JOM.0000000000001638 31348421

[casp2588-bib-0010] Caesens, G. , Stinglhamber, F. , Demoulin, S. , & De Wilde, M. (2017). Perceived organizational support and employees' well‐being: The mediating role of organizational dehumanization. European Journal of Work and Organizational Psychology, 26, 527–540. 10.1080/1359432X.2017.1319817

[casp2588-bib-0011] Colom, C. C. , & Contreras, M. J. (2018). Burnout at the supermarket: Testing the relevance of personality and stressful situations. Acción Psicológica, 15, 27–38. doi:10.5944/ap.15.2.22154

[casp2588-bib-0012] Consiglio, C. , Borgogni, L. , Vecchione, M. , & Maslach, C. (2013). Self‐efficacy, perceptions of context, and burnout: A multilevel study on nurses. La Medicina del Lavoro, 105, 255–268 PMID: 25078991. 25078991

[casp2588-bib-0013] Davy, J. A. , Kinicki, A. J. , & Scheck, C. L. (1997). A test of job security's direct and mediated effects on withdrawal cognitions. Journal of Organizational Behavior, 18, 323–349. 10.1002/(SICI)1099-1379(199707)18:4<323::AID-JOB801>3.0.CO;2-#

[casp2588-bib-0014] Dawley, D. , Houghton, J. D. , & Bucklew, N. S. (2010). Perceived organizational support and turnover intention: The mediating effects of personal sacrifice and job fit. The Journal of Social Psychology, 150, 238–257. doi:10.1080/00224540903365463 20575333

[casp2588-bib-0015] Dekker, S. W. , & Schaufeli, W. B. (1995). The effects of job insecurity on psychological health and withdrawal: A longitudinal study. Australian Psychologist, 30, 57–63. 10.1080/00050069508259607

[casp2588-bib-0016] Eagle Hill Consulting LLC . (2020). https://www.eaglehillconsulting.com/news/employee-burnout-from-covid-19-on-the-rise-with-58-of-u-s-workers-reporting-burnout/

[casp2588-bib-0017] Faul, F. , Erdfelder, E. , Lang, A. G. , & Buchner, A. (2007). G* power 3: A flexible statistical power analysis program for the social, behavioral, and biomedical sciences. Behavior Research Methods, 39, 175–191. 10.3758/BF03193146 17695343

[casp2588-bib-0018] Feather, N. T. , & Rauter, K. A. (2004). Organizational citizenship behaviours in relation to job status, job insecurity, organizational commitment and identification, job satisfaction and work values. Journal of Occupational and Organizational Psychology, 77, 81–94. 10.1348/096317904322915928

[casp2588-bib-0019] Giusti, E. M. , Pedroli, E. , D'Aniello, G. E. , Badiale, C. S. , Pietrabissa, G. , Manna, C. , … Molinari, E. (2020). The psychological impact of the COVID‐19 outbreak on health professionals: A cross‐sectional study. Frontiers in Psychology, 11, 1–9. 10.3389/fpsyg.2020.01684 32754102 PMC7366071

[casp2588-bib-0020] Hellgren, J. , & Sverke, M. (2003). Does job insecurity lead to impaired well‐being or vice versa? Estimation of cross‐lagged effects using latent variable modelling. Journal of Organizational Behavior, 24, 215–236. 10.1002/job.184

[casp2588-bib-0021] Islam, T. , Ali, G. , & Ahmed, I. (2018). Protecting healthcare through organizational support to reduce turnover intention. International Journal of Human Rights in Healthcare, 11, 4–12. 10.1108/IJHRH-03-2017-0012

[casp2588-bib-0022] Jalili, M. , Niroomand, M. , Hadavand, F. , Zeinali, K. , & Fotouhi, A. (2021). Burnout among healthcare professionals during COVID‐19 pandemic: A cross‐sectional study. MedRxiv, 94, 1345–1352. 10.1101/2020.06.12.20129650 PMC8052946 33864490

[casp2588-bib-0023] Johnson, S. J. , Holdsworth, L. , Hoel, H. , & Zapf, D. (2013). Customer stressors in service organizations: The impact of age on stress management and burnout. European Journal of Work and Organizational Psychology, 22, 318–330. 10.1080/1359432X.2013.772581

[casp2588-bib-0024] Leidner, R. (1993). Fast food, fast talk: Service work and the routinization of everyday life. Berkeley, CA: University of California Press.

[casp2588-bib-0025] Leiter, M. P. , & Maslach, C. (2016). Latent burnout profiles: A new approach to understanding the burnout experience. Burnout Research, 3, 89–100. 10.1016/j.burn.2016.09.001

[casp2588-bib-0026] Li, J. J. , Bonn, M. A. , & Ye, B. H. (2019). Hotel employee's artificial intelligence and robotics awareness and its impact on turnover intention: The moderating roles of perceived organizational support and competitive psychological climate. Tourism Management, 73, 172–181. 10.1016/j.tourman.2019.02.006

[casp2588-bib-0027] Loughnan, S. , Baldissarri, C. , Spaccatini, F. , & Elder, L. (2017). Internalizing objectification: Objectified individuals see themselves as less warm, competent, moral, and human. British Journal of Social Psychology, 56, 217–232. 10.1111/bjso.12188 28198021

[casp2588-bib-0028] Maslach, C. , Schaufeli, W. B. , & Leiter, M. P. (2001). Job burnout. Annual Review of Psychology, 52, 397–422. 10.1146/annurev.psych.52.1.397 11148311

[casp2588-bib-0052] Maslach, C. (2001). What have we learned about burnout and health?. Psychology & Health, 16, 607–611. 10.1080/08870440108405530 22804502

[casp2588-bib-0029] Mattila, A. S. , & Enz, C. A. (2002). The role of emotions in service encounters. Journal of Service Research, 4, 268–278. 10.1177/109467050200400

[casp2588-bib-0030] Mohren, D. C. , Swaen, G. M. , van Amelsvoort, L. G. , Borm, P. J. , & Galama, J. M. (2003). Job insecurity as a risk factor for common infections and health complaints. Journal of Occupational and Environmental Medicine, 45, 123–129. 10.1097/01.jom.0000052954.59271.2f 12625228

[casp2588-bib-0031] Moreno‐Jiménez, B. , Garrosa, E. , Gálvez, M. , Arias, V. , Villagrasa, J. , Rodríguez, R. , & Morante, E. (2004). Final Research Report. Analysis of Professional Burnout in the Work of Cashiers of the Community of Madrid. Madrid, Spain: Facultad de Psicología, UAM.

[casp2588-bib-0032] Morgantini, L. A. , Naha, U. , Wang, H. , Francavilla, S. , Acar, Ö. , Flores, J. M. , … Weine, S. M. (2020). Factors contributing to healthcare professional burnout during the COVID‐19 pandemic: A rapid turnaround global survey. PLoS One, 15, e0238217. 10.1371/journal.pone.0238217 32881887 PMC7470306

[casp2588-bib-0033] Nahrgang, J. D. , Morgeson, F. P. , & Hofmann, D. A. (2011). Safety at work: A meta‐analytic investigation of the link between job demands, job resources, burnout, engagement, and safety outcomes. Journal of Applied Psychology, 96, 71–94. 10.1037/a0021484 21171732

[casp2588-bib-0034] Neves, P. , & Eisenberger, R. (2014). Perceived organizational support and risk taking. Journal of Managerial Psychology, 9, 187–205. 10.1108/JMP-07-2011-0021

[casp2588-bib-0035] Oppenheimer, D. M. , Meyvis, T. , & Davidenko, N. (2009). Instructional manipulation checks: Detecting satisficing to increase statistical power. Journal of Experimental Social Psychology, 45, 867–872. 10.1016/j.jesp.2009.03.009

[casp2588-bib-0036] Probst, T. M. (2000). Wedded to the job: Moderating effects of job involvement on the consequences of job insecurity. Journal of Occupational Health Psychology, 5, 63–73. 10.1037/1076-8998.5.1.63 10658886

[casp2588-bib-0037] Ramaci, T. , Pagliaro, S. , Teresi, M. , & Barattucci, M. (2021). Job demands and negative outcomes after the lockdown: The moderating role of stigma towards Italian supermarket workers. Sustainability, 13, 1–14. 10.3390/su13137507

[casp2588-bib-0038] Rosenblatt, Z. , Talmud, I. , & Ruvio, A. (1999). A gender‐based framework of the experience of job insecurity and its effects on work attitudes. European Journal of Work and Organizational Psychology, 8, 197–217. 10.1080/135943299398320

[casp2588-bib-0039] Schaufeli, W. B. , Leiter, M. P. , Maslach, C. , & Jackson, S. E. (1996). The Maslach burnout inventory general survey. In C. Maslach , S. E. Jackson , & M. P. Leiter (Eds.), MBI manual (3rd ed., pp. 19–26). Palo Alto: Consulting Psychologists Press.

[casp2588-bib-0040] Schönbrodt, F. D. , & Perugini, M. (2013). At what sample size do correlations stabilize? Journal of Research in Personality, 47, 609–612. 10.1016/j.jrp.2013.05.009

[casp2588-bib-0041] Singh, J. (2000). Performance productivity and quality of frontline employees in service organizations. Journal of Marketing, 64, 15–34. 10.1509/jmkg.64.2.15.17998

[casp2588-bib-0042] Srivastava, S. , & Agrawal, S. (2020). Resistance to change and turnover intention: A moderated mediation model of burnout and perceived organizational support. Journal of Organizational Change Management, 33, 1431–1447. 10.1108/JOCM-02-2020-0063

[casp2588-bib-0043] Tayfur, O. , & Arslan, M. (2013). The role of lack of reciprocity, supervisory support, workload, and work‐family conflict on exhaustion: Evidence from physicians. Psychology Health & Medicine, 18, 564–575. 10.1080/13548506.2012.756535 23330970

[casp2588-bib-0044] Toppinen‐Tanner, S. , Kalimo, R. , & Mutanen, P. (2002). The process of burnout in white‐collar and blue‐collar jobs: Eight‐year prospective study of exhaustion. Journal of Organizational Behavior: The International Journal of Industrial, Occupational and Organizational Psychology and Behavior, 23, 555–570. 10.1002/job.155

[casp2588-bib-0045] Vaes, J. , & Muratore, M. (2013). Defensive dehumanization in the medical practice: A cross‐sectional study from a health care worker's perspective. British Journal of Social Psychology, 52, 180–190. 10.1111/bjso.12008 23013264

[casp2588-bib-0046] Valtorta, R. R. , Baldissarri, C. , Andrighetto, L. , & Volpato, C. (2019a). Dirty jobs and dehumanization of workers. British Journal of Social Psychology, 58, 955–970. 10.1111/bjso.12315 30706489

[casp2588-bib-0047] Valtorta, R. R. , Baldissarri, C. , Andrighetto, L. , & Volpato, C. (2019b). The dirty side of work: Biologization of physically tainted workers. International Review of Social Psychology, 32, 1–13. 10.5334/irsp.213

[casp2588-bib-0048] Vella, P. J. , Gountas, J. , & Walker, R. (2009). Employee perspectives of service quality in the supermarket sector. Journal of Services Marketing, 23, 407–421. 10.1108/08876040910985870

[casp2588-bib-0049] Volpato, C. , & Andrighetto, L. (2015). Dehumanization. In J. D. Wright (Ed.), International encyclopedia of the social & behavioral sciences (Vol. 6, pp. 31–37). Oxford: Elsevier.

[casp2588-bib-0050] Ward, J. H. (1963). Hierarchical grouping to optimize an objective function. Journal of the American Statistical Association, 58, 236–244. 10.1080/01621459.1963.10500845

[casp2588-bib-0051] Wu, L. , Rusyidi, B. , Claiborne, N. , & McCarthy, M. L. (2013). Relationships between work‐life balance and job‐related factors among child welfare workers. Children and Youth Services Review, 35, 1447–1454. 10.1016/j.childyouth.2013.05.017

