# Supplementary material for: Burnout and workplace dehumanization at the supermarket: A field study during the COVID‐19 outbreak in Italy
Source: J Community Appl Soc Psychol. 2021 Nov 29;32(4):767–85. doi: 10.1002/casp.2588 (PMC9015525; doi:10.1002/casp.2588)
Supplement: Supplementary file 1 — Data S1. Supporting information. [file CASP-32-767-s002.pdf]

Burnout and workplace dehumanization at the supermarket: A field study during the COVID-19 outbreak in Italy

Valtorta Roberta Rosa, Baldissarri Cristina & Volpato Chiara

University of Milano-Bicocca

Statement of relevance

This study explores the **psychological effects of the COVID-19 emergency on workers employed in the supermarket sector** by analysing their **levels of burnout** and the relationship between the burnout syndrome and employees' workplace experiences

A national poll conducted in April 2020 by Eagle Hill Consulting found that 45% of US essential workers reported burnout. A more severe result emerged in August 2020, when the percentage reached 58%

Our findings constitute the first empirical evidence of the presence of burnout and workplace dehumanization among supermarket employees during the COVID-19 outbreak in Italy

Theoretical background

Burnout

Dehumanization

Psychological syndrome characterized by **exhaustion, cynicism, professional inefficacy**

Theoretical background

Burnout

Dehumanization

Psychological process that refers to the idea that **people are denied their humanness** (e.g., considered as objects or viruses)

Results and conclusions

422 (82% females; 20-61 y/o) workers employed in the supermarket sector in Italy

Survey conducted between March and April 2020

- **73%** of workers had symptoms of exhaustion, cynicism, and inefficacy
- Higher **exhaustion** was associated with higher workers' perceptions of **being viewed as objects** by supervisors and customers
- **Severe burnout** was associated with higher workers' perceptions of **being viewed as viruses** by customers

- Starting point for future research to **safeguard workers' health and overall well-being** during critical times
- **Insights into the correlates of burnout and dehumanization** of frontline workers during the acute phase of the COVID-19 pandemic
