# Supplementary material for: Burnout and workplace dehumanization at the supermarket: A field study during the COVID‐19 outbreak in Italy
Source: J Community Appl Soc Psychol. 2021 Nov 29;32(4):767–85. doi: 10.1002/casp.2588 (PMC9015525; doi:10.1002/casp.2588)
Supplement: Supplementary file 2 — Data S2. Supporting information. [file CASP-32-767-s001.pdf]

Burnout e deumanizzazione nei supermercati: Uno studio tra i lavoratori durante la pandemia di COVID-19 in Italia

Valtorta Roberta Rosa, Baldissarri Cristina & Volpato Chiara

Università degli Studi di Milano-Bicocca

Dichiarazione di rilevanza

Questo studio indaga **gli effetti psicologici dell'emergenza di COVID-19 sui lavoratori impegnati nei supermercati italiani** esplorando i **livelli di burnout** e la relazione tra questa sindrome e le esperienze dei dipendenti sul posto di lavoro

Un sondaggio condotto nell'aprile 2020 da Eagle Hill Consulting ha rilevato che il 45% dei lavoratori americani impegnati nel settore alimentare ha riportato burnout. Un risultato più grave è emerso nell'agosto 2020, quando la percentuale ha raggiunto il 58%

I nostri risultati forniscono una prima evidenza empirica della presenza di burnout e deumanizzazione lavorativa tra i dipendenti dei supermercati italiani durante l'epidemia di COVID-19

Quadro teorico

Burnout

Deumanizzazione

Sindrome psicologica caratterizzata da **esaurimento, cinismo, inefficacia professionale**

Quadro teorico

Burnout

Deumanizzazione

Processo psicologico che prevede la **negazione di umanità degli altri** (considerati, ad esempio, come oggetti e virus)

Risultati e conclusioni

422 (82% donne; 20-61 anni) lavoratori impegnati in diversi supermercati italiani

Indagine svolta tra marzo e aprile 2020

- **73%** dei lavoratori ha riportato sintomi di esaurimento, cinismo e inefficacia professionale
- Alti livelli di **esaurimento** sono associati a una maggiore percezione dei lavoratori di essere **considerati come oggetti** dai superiori e dai clienti
- Alti livelli di **burnout** sono associati a una maggiore percezione dei lavoratori di essere **considerati come virus** dai clienti

- Punto di partenza per ricerche future finalizzate alla **tutela della salute e del benessere generale dei lavoratori** durante i periodi critici
- **Nuovi spunti di riflessione sulla relazione tra burnout e deumanizzazione** dei lavoratori in prima linea durante la fase acuta della pandemia di COVID-19
